# Copper Chalcogenide–Copper Tetrahedrite Composites—A New Concept for Stable Thermoelectric Materials Based on the Chalcogenide System

**DOI:** 10.3390/ma14102635

**Published:** 2021-05-18

**Authors:** Andrzej Mikuła, Krzysztof Mars, Paweł Nieroda, Paweł Rutkowski

**Affiliations:** Faculty of Materials Science and Ceramics, AGH University of Science and Technology, Al. Mickiewicza 30, 30-059 Krakow, Poland; kmars@agh.edu.pl (K.M.); pnieroda@agh.edu.pl (P.N.); pawelr@agh.edu.pl (P.R.)

**Keywords:** composite, copper chalcogenide, tetrahedrite, thermoelectrics

## Abstract

For the first time, an alternative way of improving the stability of Cu-based thermoelectric materials is proposed, with the investigation of two different copper chalcogenide–copper tetrahedrite composites, rich in sulfur and selenium anions, respectively. Based on the preliminary DFT results, which indicate the instability of Sb-doped copper chalcogenide, the Cu_1.97_S–Cu_12_Sb_4_S_13_ and Cu_2−x_Se–Cu_3_SbSe_3_ composites are obtained using melt-solidification techniques, with the tetrahedrite phase concentration varying from 1 to 10 wt.%. Room temperature structural analysis (XRD, SEM) indicates the two-phase structure of the materials, with ternary phase precipitates embed within the copper chalcogenide matrix. The proposed solution allows for successful blocking of excessive Cu migration, with stable electrical conductivity and Seebeck coefficient values over subsequent thermal cycles. The materials exhibit a p-type, semimetallic character with high stability, represented by a near-constant power factor (PF)—temperature dependences between individual cycles. Finally, the thermoelectric figure-of-merit ZT parameter reaches about 0.26 (623 K) for the Cu_1.97_S–Cu_12_Sb_4_S_13_ system, in which case increasing content of tetrahedrite is a beneficial effect, and about 0.44 (623 K) for the Cu_2−x_Se–Cu_3_SbSe_3_ system, where increasing the content of Cu_3_SbSe_3_ negatively influences the thermoelectric performance.

## 1. Introduction

**Figure 1:**
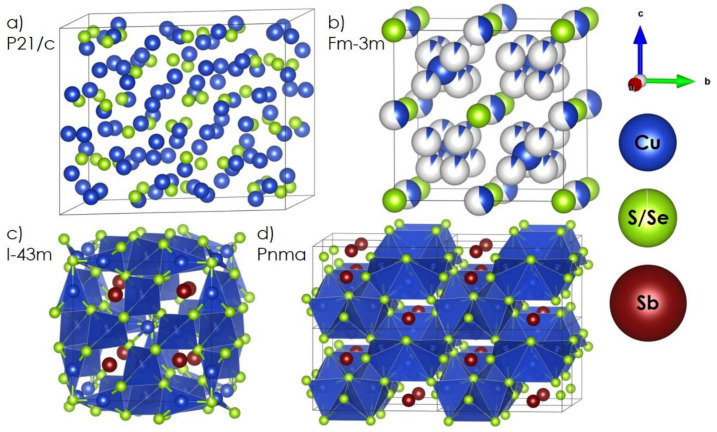
Schematic representation of the considered structures: (**a**) monoclinic, low-temperature Cu_2−x_Ch, (**b**) cubic, high-temperature Cu_2−x_Ch (considering partially occupied sites), (**c**) cubic tetrahedrite, (**d**) orthorhombic Cu_3_SbCh_3_.

In the era of global energy and the climate crisis, the development of low-pollution energy-conversion technologies constitutes one of the top priorities among the scientific community. Transition metal chalcogenides, especially copper-based ones, including copper (I) sulfide and copper (I) selenide, are among the most prominent and extensively investigated materials in this context, offering multiple functional properties that can be used in several potential applications such as photoelectrochemical, photocatalytic, or solar cells [1,2,3,4], as well as, primarily, thermoelectrics [5,6,7]. Copper chalcogenides with a general formula of Cu_2−x_Ch (where x varies from 0 to 0.2) are often classified as so-called superionic conductors, the unique transport properties of which can be described on the basis of the phonon-liquid electron-crystal (PLEC) theory [5,8]. While a vast number of different structural and compositional substructures can be distinguished in Cu_2−x_Ch systems, high-temperature, highly symmetrical cubic structures (Figure 1a,b, phase transition at about 600 K for both Cu_2−x_S and Cu_2−x_Se, respectively) are by far the most interesting with regard to energy-conversion technologies. They are characterized by the high diffusivity values of copper ions resulting from multiple structural positions, between which the copper ions can jump in short time periods, while low-mobility chalcogenide ions create a crystalline pathway for charge carriers. Additionally, the high mobility of Cu ions introduces extra scattering within the crystal structure and significantly shortens free paths for lattice phonons, directly affecting heat transport. For these reasons, Cu_2−x_Ch materials possess high electrical conductivity, very low thermal conductivity, and relatively high values of Seebeck coefficient [9], reaching enormous ZT thermoelectric figure-of-merit parameters (given by Equation (1)) at the level of 2–2.5 (900 K) [8],

(1) $$ZT=\frac{\alpha^{2}\sigma T}{\lambda }$$

where *ZT*—thermoelectric figure-of-merit, *α*—Seebeck coefficient, *σ*—electrical conductivity, *λ*—thermal conductivity, *T*—temperature.

Considering the extremely high performance of copper chalcogenides, it must be noted that their stability under operating conditions is relatively low, mainly due to the aforementioned high mobility of copper ions [5], often disqualifying them from practical use. As described by Brown et al. [10] and Dennler et al. [11], the directional diffusion of Cu ions (parallel to the temperature or current gradient) leads to the precipitation of free copper at the surface of the samples and, consequently, to a significant worsening of the transport properties, even up to 40% for stoichiometric Cu_2_S [12]. Nevertheless, the vast majority of studies in this field are still mainly devoted to maximizing ZT, neglecting the long-term stability issue. As indicated by recent advances, the stability of Cu_2−x_Ch can be improved by precise control of stoichiometry, particularly by adjusting the number of Cu vacancies [12], incorporating nano-inclusions [13] or through doping processes [14]. In the latter case, both cationic and anionic sublattices can be considered. The most common solution is doping of the cation lattice sites with less mobile, and/or electron donor ions [15,16,17], which can influence both the stability and the conductivity type, potentially allowing n-type semiconductors to be obtained. Due to significantly limited availability of these, it should be perceived as an additional benefit. However, as indicated by one of our previous studies on the example of iron dopant, the effectiveness of such a method can be inadequate, as the cyclic nature of thermoelectric operation leads to the exclusion of Fe additions from the structure over time [18,19]. The doping of the anionic sublattices may be another viable solution, as shown by Zhang et al., who obtained Te-doped copper selenide characterized by excellent stability. However, this came at the cost of inferior performance, indicated by ZT at a level of 0.35 (650 K), [14]. Consequently, other means of improving stability are still being pursued. An extremely promising idea in terms of blocking Cu migration was recently presented by Yang et al. [20], who synthesized a composite based on the copper selenide and BiCuSeO oxyselenide nanoparticles. The obtained material was characterized by ZT parameter at a level of 2 (1000 K), with proven suppression of copper ions migration and only limited precipitation of free copper.

Considering the recent scientific achievements in the field of doped copper chalcogenides, the main objective of this study was to investigate the possibility of modifying the properties of these materials by introducing immobile Sb ions to the system. This issue was tackled on both theoretical and experimental grounds, mainly with respect to their long-term stability under cycling thermal conditions. As will be shown, based on the theoretical analysis, the most promising way of addressing the discussed stability issues throguh Sb modification is to design composite systems of Cu_2−x_Ch and another stable (energy-beneficial, see the results) copper-based, Sb-rich chalcogenide, in this case tetrahedrite-based, structure. Copper tetrahedrites Cu_12_Sb_4_Ch_13_ (naturally occurring minerals [21], Figure 1c), and very similar ternary diamond-like chalcogenides with the general formula varying from Cu_3_SbCh_3_ to Cu_3_SbCh_4_ (Figure 1d), are another type of Cu-based thermoelectrics. They are characterized by the presence of Sb ions in structural cages, which leads to the part-crystalline, part-liquid state of the Cu ions [22], resulting in ultra-low lattice thermal conductivity λ_L_ [5]. In the form of solid solution or doped phases, these ternary Cu-Sb-Ch materials are characterized by good stability due to Sb–Ch bonds being significantly stronger compared to Cu–Ch ones, and can reach ZT parameter levels from 0.6 up to 1.2 for tetrahedrites [5,23,24,25,26], and about 0.4–0.9 for Cu_3_SbCh_4-x_ compounds [5,27,28,29]. Due to the high-temperature degradation of these phases, their applications are limited to temperatures up to 700. Assuming analogous mechanisms to those presented by Yang et al. [20], and considering the results of our previous study [19], according to which the presence of a secondary, structurally similar phase creates a diffusion barrier and limits the excessive migration of copper ions from Cu_2−x_Ch, a composite material with improved stability should be attainable. Therefore, the presented work focuses on the possibility of obtaining copper chalcogenide–copper tetrahedrite composites and investigating their thermoelectric and stability performance.

## 2. Materials and Methods

Ab initio calculations addressing the preliminary estimation of the stability of the considered systems were carried out using the Wien2k package [30], employing Density Functional Theory formalism (DFT) and the Full Potential Augmented Plane Waves (FP-LAPW) method. The computations were performed for the cubic tetrahedrite structures (Cu_12_Sb_4_S_13_, and Cu_12_Sb_4_Se_13_) and 2 × 2 × 2 supercells (in order to allow the investigation of relatively small amounts of Sb dopant), preserving the high-temperature, cubic form of Cu_2_S and Cu_2_Se. The considered Sb-doped model structures took into account the possibility of Sb dopants occurring in different structural positions (Cu initial sites), with concentration varying from 1.5 to 12.5 mol. %. The presence of Cu vacancies was also recognized. As the indicator of the thermodynamic stability of individual structures, the defect formation energies calculated in accordance with Equation (2) were investigated:

(2) $$E^{f}\left[Sb\right]=E_{total}\left[Cu_{2}Ch+Sb\right]-E_{total}\left[Cu_{2}Ch\right]-E\left[Sb\right]+E\left[Cu\right]$$

where

*E^f^* [*Sb*] is the single defect formation energy for Sb substituting Cu atom in Cu_2_Ch structure;
*E_total_* [*Cu_2_S* + *Sb*] is the total energy of the structure with Sb dopant at Cu initial site;
*E_total_* [*Cu_2_S*] is the total energy of the bulk Cu_2_Ch structure;
*E* [*Sb*]—energy of Sb atom;
*E* [*Cu*]—energy of Cu atom.

Defect formation energies were calculated by implementing the enthalpy of creation approach, which predicts the decomposition of the starting materials into the most thermodynamically stable phases of particular elements, in this case: Cu—cubic Fm-3 m, Sb—rhombohedral R-3 m, S—orthorhombic Fddd, and Se—trigonal P3121, respectively. For the pure-phase systems, namely Cu_2_Ch and tetrahedrites, the enthalpy of creation was also calculated in accordance with the same principles, using Equations (3) and (4) for Cu_2_Ch and Cu_12_Sb_4_Ch_13_, respectively:

(3) $$\Delta H\;\left[Cu_{2}Ch\right]=E_{total}\left[Cu_{2}Ch\right]-2E\left[Cu\right]-E\left[Ch\right]$$

(4) $$\Delta H\;\left[Cu_{12}Sb_{4}Ch_{13-x}\right]=E_{total}\left[Cu_{12}Sb_{4}Ch_{13-x}\right]-12E\left[Cu\right]-\left(13-x\right)E\left[Ch\right]-4E\left[Sb\right]$$

where

Δ*H* is the enthalpy of formation;
*E_total_* is the total energy of the bulk structure;
*E* [*Cu*]—energy of Cu atom;
*E* [*Sb*]—energy of Sb atom;
*E* [*Ch*]—energy of S atom.

For all calculations, the following computational criteria were used: exchange-correlation potential PBEsol [31] with spin-polarization mode, Rkmax = 7.0, Gmax = 14.0, and k point mesh proportional to the size of an irreducible Brillouin zone.

For the experimental part of the study, high-purity elements in the form of powders (Cu—99.9% Alfa Aesar, Sb—99.5% Alfa Aesar Chemicals, Haverhill, MA, USA) and pieces (S—99.999% Alfa Aesar, Se—99.999%) were weighted according to the nominal compositions presented in Table 1, and double sealed in quartz ampoules under vacuum conditions (10^−3^ atm). After initial homogenization, the final materials were synthesized by using the melt-solidification technique in a tube furnace. Firstly, the ampules were heated to 573 K (5 K·min^−1^) and annealed for 12 h in order to carry out the initial reaction between liquid sulfur (below boiling point) and other elements. Next, the temperature was increased to 1423 K (1 K·min^−1^), followed by a 96 h annealing period. Finally, the furnace was cooled to room temperature (RT) at 5 K·min^−1^. Attempts to quench the high-temperature phases from 1173 K and 773 K, commonly reported for Cu_2_Ch compounds [17,32], failed, as the final products indicated the mix of Cu-Ch phases, due to disturbed ratio of Cu and Ch, and precipitation of free Sb or Sb_2_Ch_3_-based phases. Due to the high density of the obtained ingots (5.39–5.48 g·cm^−3^ for sulfur-rich composites, and 6.57–6.70 g·cm^−3^ for selenium-rich ones, as determined by Archimedes’ principle, Table 1), and close-to-optimal geometry (cylinder with a diameter of about 10 mm), the samples for structural, electrical, and thermal studies were cut directly from the obtained ingots without additional processing (e.g., sintering).

Phase compositions of the prepared samples were examined by means of X-ray diffraction (XRD) (apparatus: Empyrean PANalytical (CuKα radiation, Malvern Panalytical, Worcestershire, UK), measurements were conducted in the range of 10–90 2Θ with step 0.008°), and further assessed with the use of X’Pert High Score software (Malvern Panalytical, Worcestershire, UK). The microstructure and homogeneity of the samples were investigated by scanning electron microscopy combined with energy-dispersive X-ray spectroscopy (SEM + EDS) (apparatus: NOVA NANO SEM 200, FEI COMPANY, Hillsboro, OR, USA) acceleration voltage equal to 18 kV, equipped with EDAX analyzer).

Electrical conductivity and Seebeck coefficient measurements were performed by means of a self-designed DC four-point van der Pauw method in the 293–623 K temperature range. These dynamic measurements were carried out under Ar atmosphere with temperature step 25°. The temperature was stabilized by 35 min annealing at each measurement point. For Seebeck coefficient measurements, the temperature gradient between the hot and cold side was set to 3°. The stability of the materials was investigated on the basis of these measurements, conducted over three subsequent heating and slow-cooling cycles.

Thermal conductivity coefficient λ was determined (Equation (5)) on the basis of sample densities (Table 1); thermal diffusivity κ and specific heat C_p_ measurements were carried out with the use of the NETZSCH LFA 427 (laser flash analysis, Ar atmosphere, Netzsch group, Selb, Germany) and NETZSCH STA 449 F3 thermal analyzer (Netzsch group, Selb, Germany), respectively, in the same temperature range of 293–623 K.

(5) $$\lambda =\kappa C_{p}\rho$$

## 3. Results and Discussion

### 3.1. Defect Formation Energy

**Figure 2:**
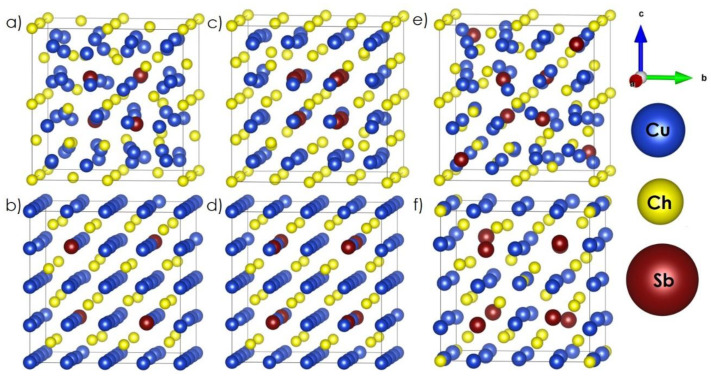
Optimized model structures: (**a**) Cu_1.88_Sb_0.12_Ch_v1, (**b**) Cu_1.88_Sb_0.12_Ch_v2, (**c**) Cu_1.75_Sb_0.25_Ch_v1, (**d**) Cu_1.75_Sb_0.25_Ch_v2, (**e**) Cu_1.75_Sb_0.25_Ch_v3, (**f**) Cu_12_Sb_4_Ch_13_.

In the first part of the study, the possibility of doping the Cu_2_Ch structures with immobile Sb ions was assessed on the basis of theoretical analysis. In Figure 2, the optimized Sb-doped Cu_2_Ch, differing with respect to Sb dopant location sites (Figure 2a–e) and tetrahedrite (Figure 2f) model structures, is presented, with increasing content of Sb. In the main article, only the Cu_1.88_Sb_0.12_Ch (Figure 2a,b) and Cu_1.75_Sb_0.25_Ch (Figure 2c–e) compositions are presented, as they depict the case where substitution of Cu ions by Sb ones is possible without distorting the cubic symmetry. In the case of Cu_1.88_Sb_0.12_Ch, two possible distributions of Sb dopants can be distinguished, with the next Sb ions existing either in the first (Figure 2a assigned as Cu_1.88_Sb_0.12_Ch_v1) or the second (Figure 2b assigned as Cu_1.88_Sb_0.12_Ch_v2) coordination sphere in relation to the selected one. In both cases, the Sb ions are arranged in alternation with Cu ions (4e Wyckoff position). For Cu_1.75_Sb_0.25_Ch, three possible distributions of Sb can be distinguished, where Sb ions exist close to each other in the first coordination sphere (Figure 2c assigned as Cu_1.75_Sb_0.25_Ch_v1), in the second coordination sphere in relation to each other, and alternately with Cu ions (Figure 2d assigned as Cu_1.75_Sb_0.25_Ch_v2), and as a combination of positions in the first and second coordination spheres in relation to each other and alternately with Cu ions (Figure 2e assigned as Cu_1.75_Sb_0.25_Ch_v3). The rest of the optimized structures can be found in the Supplementary Materials (Figure S1, S- or Se-rich structures differ in the unit cell parameters only). A summary of all of the considered compositions is presented in Table 2. It is clear that when Sb dopants occur relatively close to each other, the remaining ions move away from the Sb ones, causing significant distortion and creating more open space for antimony ions (Figure 2a,c,e). On the other hand, when Sb ions take alternate positions with regard to Cu and are relatively far from each other (Figure 2b,d), the structural distortions are significantly smaller. Larger distances between individual Sb dopants, combined with an alternating arrangement of Sb and Cu ions, are also more energetically beneficial, as indicated by the defect formation energies given in Table 2, particularly for Se-rich structures, characterized by larger unit cell parameters (11.266 Å, and 11.538 Å for Cu_1.88_Sb_0.12_S_v2 and Cu_1.75_Sb_0.25_S_v3; 11.680 Å, and 11.785 Å for Cu_1.88_Sb_0.12_Se_v2 and Cu_1.75_Sb_0.25_Se_v3, respectively). It can also be noticed that the most energy-favorable arrangement in the Sb-doped system occurs when Sb ions are placed as far as possible from each other (Figure 2b for small dopant concentration, and Figure 2e for greater concentration), under the assumption that any Sb dopant tends to repel other ions from itself. The model structures where the Sb ions have a higher degree of separation from each other or exist in symmetrical sites (without Cu ions between them, Figure 2c,d), are much less disturbed and are characterized by significantly lower cell parameters, which should be considered as energetically unfavorable.

Additionally, the structures where Sb substitutes Cu as a heterovalent dopant within a small concentration range (Table 2), with simultaneous creation of Cu vacancies, are also characterized by negative or close-to-zero values of defect formation energy. Such a behavior seems to be justified by a much easier means of creating more open space for Sb ions. It can therefore be concluded that the energy-beneficial effects include Sb and Cu ions in alternate arrangements, Sb dopants occurring as far as possible from each other, and Sb dopants existing in open space sites with simultaneous creation of Cu vacancies and/or pushing other ions from themselves. Keeping the above observations in mind, it can be noticed that if some of the Cu ions are removed from the model structure presented in Figure 2d, the obtained one will represent the slightly distorted structure of tetrahedrite (Figure 2f), where the positions of antimony ions meet all requirements identified as energy-beneficial. Thus, it is strongly suggested that Sb-doped Cu_2_Ch systems would seek a way to form a ternary type of structure, characterized by negative values of enthalpy (Table 2). The described observations are the first indicators that doping Cu_2_Ch by Sb ions, both as homo- and heterovalent dopant, cannot be successful unless a limited solubility of Sb occurs, within which it can be introduced into the structure. Cu_2_Se structures can be identified as the more likely host, due to the significantly larger unit cells. On the other hand, the most energy-favorable phases are Cu_2−x_Ch (where x varies from 0 to 0.2), as indicated by the negative energy value of a single Cu vacancy, and the ternary phase (Cu_12_Sb_4_Ch_13_). Thus, the co-existence of these phases (composite materials) as a stable system is specifically possible, and our main objective, namely, reducing excessive Cu migration, can be achieved by obtaining composite materials, rather than single-phase, Sb-doped ones.

### 3.2. Structural Properties and Microstructure Observations

**Figure 3:**
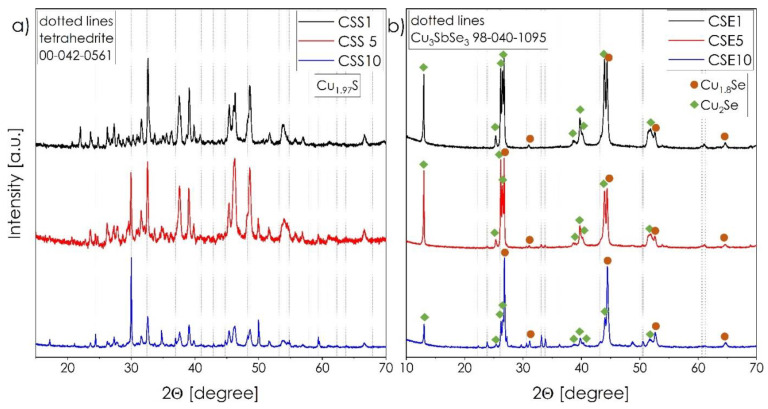
XRD patterns of the Cu_1.97_S–Cu_12_Sb_4_S_13_ (**a**) and Cu_2−x_Se–Cu_3_SbSe_3_ (**b**) composites.

Based on the results of theoretical analysis, selected composite materials comprised of copper chalcogenide and copper tetrahedrite phases, with the nominal compositions presented in Table 1, were synthesized. The room-temperature X-ray diffraction patterns recorded for the composite systems are presented in Figure 3. In the case of sulfur-rich samples (Figure 3a), the presence of orthorhombic Cu_1.97_S as a primary phase and the tetrahedrite Cu_12_Sb_4_S_13_ characterized by cubic symmetry as a secondary phase, can be observed. The presence of the orthorhombic Cu_1.97_S phase instead of the Cu_2_S can be explained by the natural tendency towards creating copper vacancies in Cu_2−x_S (the formation of Cu vacancy defects in both considered Cu_2−x_Ch materials is an energy-favorable effect, Table 2), with a simultaneous tendency for non-stoichiometry in the tetrahedrite’s anionic sublattice. The orthorhombic phase, as shown by Zhao et al. [12], is also characterized by much superior properties, both in terms of stability and thermoelectricity than the monoclinic Cu_2_S one. Therefore, its presence should be considered a beneficial effect. Lastly, it is worth noting that the main phase does not appear to be influenced by the increasing amount of ternary phase.

The phase structure of the Se-based series of materials, however, exhibits a much greater variety (Figure 3b). Here, the mixture of two binary Cu_2−x_Se compounds, namely cubic Cu_2_Se and cubic Cu_1.8_Se, is the dominating one, with the addition of ternary orthorhombic, diamond-like Cu_3_SbSe_3_ as a secondary phase. The increased amount of Cu_3_SbSe_3_ influences the Cu_2_Se and Cu_1.8_Se ratio with the increase of the latter’s content. Based on the theoretical studies, the Cu_1.8_Se should be more stable in the cubic form than the Cu_2_Se, while the presence of Cu_12_Sb_4_Se_13_ allows for accommodating the excessive Cu ions within the ternary phase. Further refinement of the nominal composition, based on the experimentally identified phases, did not enable a reduction in the number of occurring phases, showing that energy-wise, the coexistence of all phases is beneficial.

**Figure 4:**
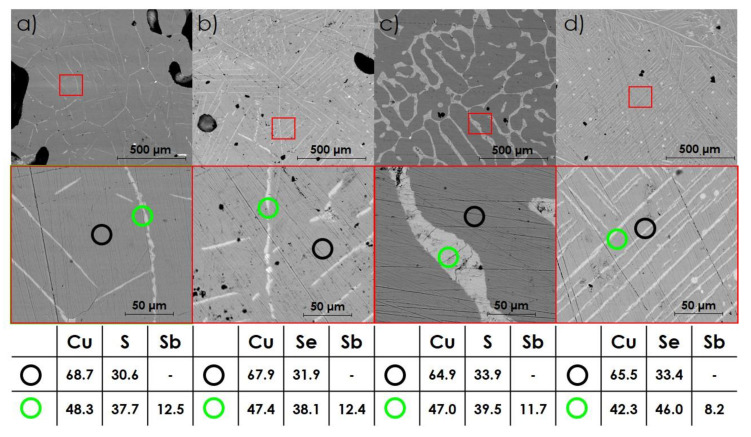
SEM micrographs together with the corresponding EDS point analysis (% at.): (**a**) CSS5, (**b**) CSS10, (**c**) CSE5, (**d**) CSE10. All data were collected from the fractured as-synthesized ingot.

SEM observations presented in Figure 4 confirm the presence of composite materials with ternary, randomly oriented phases embedded in the form of fibers or plates in Cu_2−x_Ch matrix. Due to the minor precipitation of the secondary phase, the SEM micrographs of CSS1 and CSE1 samples are not included. In both S- and Se-rich compositions, the observed amount of Sb-containing phases increases according to the nominal ratios of phases. Regardless of the main symmetry of the Cu-Sb-Ch phases determined on the basis of XRD results, the precipitation of these compounds occurs at the grain boundaries, which is particularly evident in Figure 4a,c characterized by 5 wt.% addition of Sb-rich phases. Based on SEM observations, however, two similar Cu_2−x_Se phases, namely Cu_2_Se and Cu_1.8_Se, cannot be distinguished. The quantitative EDS analysis in the selected points indicates the chemical composition close to the assumed and agrees well with the XRD results.

### 3.3. Transport Properties

**Figure 5:**
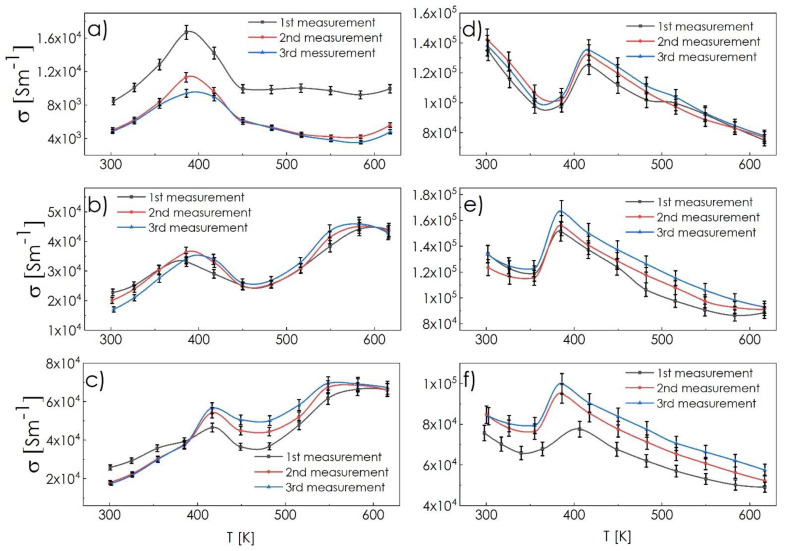
Cyclic measurements of electrical conductivity as a function of temperature recorded for the composite ingots: (**a**) CSS1, (**b**) CSS5, (**c**) CSS10, (**d**) CSE1, (**e**) CSE5, (**f**) CSE10.

During the design of composite materials for thermoelectric applications, the relative stability of each component must be taken into account. As indicated by the previous authors, cyclic measurements above 700 K may result in degradation of ternary chalcogenide structures, which may, in turn, lead to the partial incorporation of the Sb dopant into the high-temperature cubic form of Cu_2−x_Ch (as indicated by defect formation energies for a small amount of Sb, Table 2), and its subsequent removal during the cooling process. Such a behavior has been observed, e.g., in copper chalcogenide–bornite structures [19]; thus, the cyclic measurements in this study were conducted in the temperature range up to 650 K. In Figure 5, the results of the total electrical conductivity measurements as a function of temperature are presented. High electrical conductivity, at the level of 10^4^ and 10^5^ Sm^−1^, for sulfur-rich (Figure 5a–c) and selenium-rich (Figure 5d–f) composites, respectively, was observed, which is comparable to the results for the phase-pure Cu_2−x_Ch compounds [12,33,34]. Additionally, the phase transitions at about 400 and 600 K (into hexagonal, and cubic form, respectively), typical for low-symmetrical Cu_2−x_S compounds [12,32,35], can be distinguished. Clear tendencies to shift these transitions into lower and higher temperature ranges, for the first- and second-phase transition, respectively, can be noticed in the CSS systems. These changes are accompanied by the increasing values of total electrical conductivity with higher content of tetrahedrite phase, which should be viewed as a synergistic effect of these two phases’ co-existence (pure-phase tetrahedrite is characterized by similar or slightly lower electrical conductivity in comparison to Cu_2−x_S [23,36,37]). Among the considered materials, only CSS1 sample exhibits a significant decrease of recorded conductivity values over subsequent cycles, which is most probably related to the dominant Cu_1.97_S-like character of this sample. On the other hand, CSS5 and CSS10 possess high stability in this regard, and a positive influence of successive cycles on the conductivity value can even be observed. Such a positive effect can also be recognized for the CSE series of samples. On the other hand, in their case, the presence of the additional ternary phase causes lowering of the total electrical conductivity with the increasing content of Cu_3_SbSe_3_ phase, which is in agreement with the literature data, as diamond-like compounds usually possess lower electrical conductivity in comparison to Cu_2−x_Se [27,38]. The total values of electrical conductivity at the level of 10^5^ sm^−1^, with a clear peak at about 400 K, are typical for Cu_2−x_Se chalcogenides with some non-stoichiometry in the cationic sublattice [33,34]. The changing position of this maximum in Figure 5d–f also corresponds to the order–disorder transition of the Cu_3_SbSe_3_ [38], which can overlap with the aforementioned phase transition of Cu_2−x_Se. Some differences in electrical conductivity between successive cycles can also be related to the relaxation processes.

**Figure 6:**
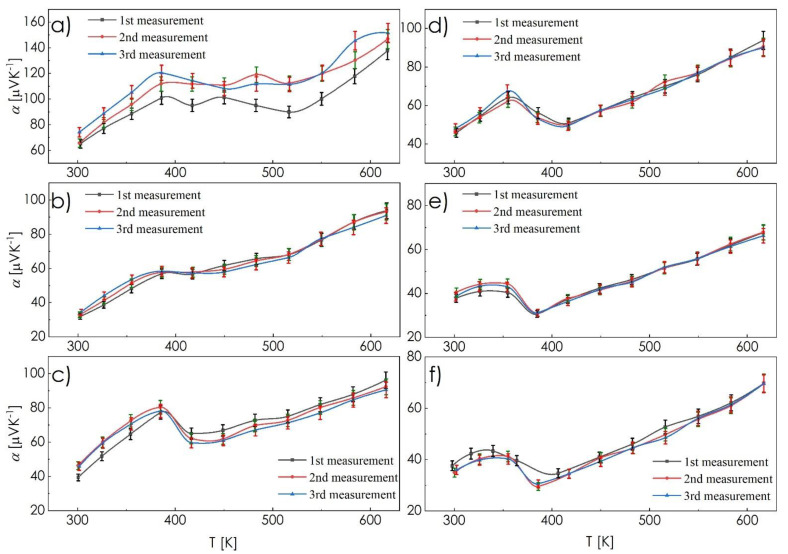
Cyclic measurements of the Seebeck coefficient as a function of temperature recorded for composite ingots: (**a**) CSS1, (**b**) CSS5, (**c**) CSS10, (**d**) CSE1, (**e**) CSE5, (**f**) CSE10.

Figure 6 shows the results of the cyclic Seebeck coefficient measurements as a function of temperature. Here, in contrast to the electrical conductivity results, the thermopower values as a function of temperature are almost constant between successive cycles for all studied compositions, excluding CSS1 sample (Figure 6a), where a clear difference between the first and subsequent cycles can be observed. For the latter sample, the Seebeck coefficient slightly increases after the first cycle, which is in accordance with the results of the electrical conductivity measurements of this ingot, suggesting changes in charge carrier concentration (increasing thermopower and decreasing conductivity) that may also be related to the dominating influence of Cu_1.97_S phase and its Cu ions migration, with an insufficient amount of tetrahedrite phase to stop it.

When analyzing the amount of ternary phase in the presented materials, one can conclude that its increasing concentration negatively affects the Seebeck coefficient. Individual components of considered composites, namely Cu_2−x_S, Cu_2−x_Se, Cu_12_Sb_4_S_13_, and Cu_3_SbSe_3_ phases, as phase-pure compounds, indicate similar and positive (p-type, [12,21,34,36,38]) values of Seebeck coefficient at the levels of Cu_1.97_S—100–200 μVK^−1^ [12], Cu_2−x_Se—20–60 μVK^−1^ for stoichiometric Cu_1.8_Se, and 70–150 μVK^−1^ for Cu_2−x_Se, Cu_12_Sb_4_S_13_—120–170 μVK^−1^ [37], and Cu_3_SbSe_3_—80–200 μVK^−1^ [38], respectively (the minimum and maximum values in each range correspond to the increasing temperature). Similar but not identical values of thermopowers may result in the creation of micro-cell pairs, composed of two phases with different Seebeck coefficients, thus inducing the presence of eddy currents and lowering the total Seebeck coefficient. Here, even CSS1 and CSE1 samples with the dominant influence of binary phases, are characterized by lower thermopower values in comparison to bulk Cu_1.97_S and Cu_2−x_Se. Further increase of the content of ternary phases causes a subsequent lowering of the Seebeck coefficient. Such effects are the reason the most common approach to the design of thermoelectric materials focuses on phase-pure materials. The presented results, however, indicate an alternative way of producing thermoelectric materials with improved stability, even at the cost of their performance, allowing for near-constant values of transport properties as a function of temperature between subsequent cycles.

**Figure 7:**
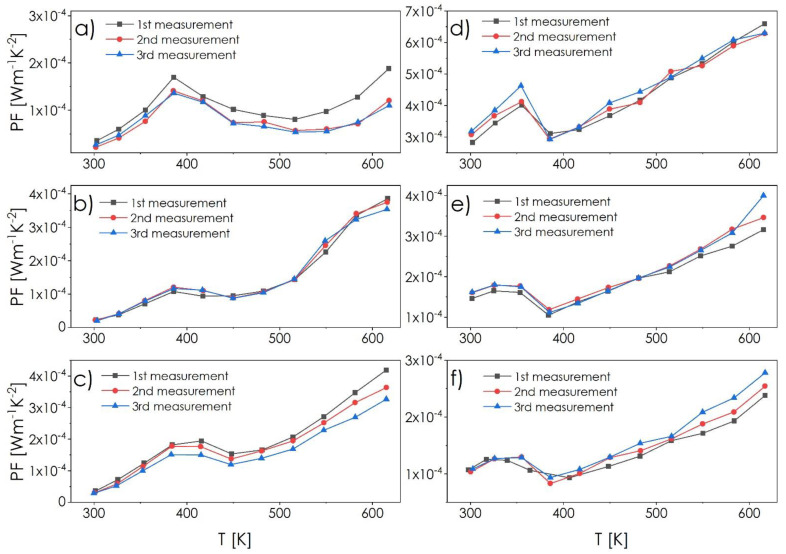
Power factor as a function of temperature determined on the basis of conductivity and Seebeck coefficient measurements (PF = σα^2^) for composite ingots: (**a**) CSS1, (**b**) CSS5, (**c**) CSS10, (**d**) CSE1, (**e**) CSE5, (**f**) CSE10.

Based on the electrical conductivity and Seebeck coefficient measurements, the Power Factor (PF = σα^2^) was determined as a function of temperature, with the results being summed up in Figure 7. For all samples, highly repeatable values at the level of 10^−4^ W·m^−1^·K^−2^ are visible, with a slight tendency towards lowering over subsequent cycles for the CSS series, and an opposite one for the CSE series. These changes are, however, negligible in relation to the results typically reported for the pure-phase Cu_2−x_Ch compounds, which exhibit profound degradation of properties during cycling, and prove the stability of composite systems, representing the most valuable achievement of this work.

**Figure 8:**
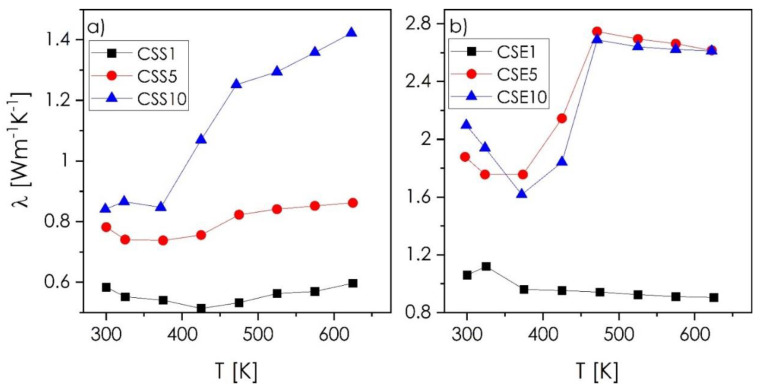
Thermal conductivity as a function of temperature recorded for sulfur-rich (**a**) and selenium-rich (**b**) composite ingots.

**Figure 9:**
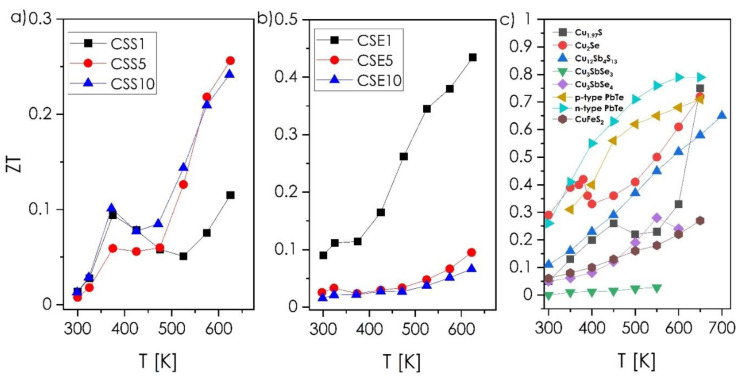
ZT—thermoelectric figure-of-merit parameter as a function of temperature determined for sulfur-rich (**a**), and selenium-rich composite ingots (**b**) in comparison to typical chalcogenide materials (**c**); [5,9,38,39,40,41,42] and references therein.

The results of the thermal conductivity measurements, presented in Figure 8, turned out to be rather surprising. The phase-pure components of the studied systems are reported to exhibit the following values of the total thermal conductivity (varying with increasing temperature): Cu_1.97_S—0.5–0.4 W·m^−1^·K^−1^ [12], Cu_1.8_Se—5.0–3.0 W·m^−1^·K^−1^, Cu_2−x_Se—2.0–1.0 W·m^−1^·K^−1^ [33,34], Cu_12_Sb_4_S_13_—0.6–1.0 W·m^−1^·K^−1^ [21,37], and Cu_3_SbSe_3_—0.6–0.3 W·m^−1^·K^−1^ [38]. Based on these values, it was expected that the presence of the ternary phase would, if not reduce the thermal conductivity values, at least allow them to be maintained at a level similar to those known for Cu_2−x_Ch. Indeed, as assumed, the CSS1 and CSE1, dominated by the Cu_2−x_Ch phases, possess thermal conductivity values comparable to Cu_1.97_S and Cu_2−x_Se, respectively. However, the increasing content of the ternary phase, characterized by the inferior thermal conductivity, as mentioned above, leads to a significant increase of thermal conductivity up to 1.4 W·m^−1^·K^−1^ for CSS10 and about 2.7 W·m^−1^·K^−1^ for CSE10 (600 K), clearly exhibiting properties beyond the rule of mixtures. Based on the presented electrical conductivity, Seebeck coefficient, and thermal conductivity results, it can be postulated that, macroscopically, the presence of ternary phases at the grain boundaries in the Cu-Sb-S system leads to enhancing metallic character of the materials. In the case of the Cu-Sb-Se system, a significant drop of the thermal conductivity (373 K) can be observed due to aforementioned phase transition and the so-called critical phenomena of Cu_2−x_Se [5,39]. Further increase of thermal conductivity is related to the presence of the ternary phase, which significantly reduces the reflection of the lattice phonons between neighboring grains. As a result, the ternary phase creates an easy pathway for heat flow. The same mechanism was noticed by Bailey et al. for the Cu_2_Se–SnSe system [39]. This mechanism, combined with increased metallic character, is also related to the CSS sample series. While beneficial to the electrical conductivity, such a behavior has a strongly negative impact on the thermal conductivity, and consequently, on the final thermoelectric figure-of-merit values (Figure 9a,b). Here, the ZT values calculated for CSS1 and CSE1 samples are at a comparable level to the pure-phase Cu_2−x_Ch compounds (in the considered temperature range, Figure 9c). For the compositions with higher contents of ternary phase, its increasing amount led to the increase of the ZT in the case of CSS series, and the decrease of the ZT for CSE series. Finally, the highest ZT parameter reaches values of 0.26 for the CSS5 and 0.44 for the CSE1 composition, respectively (623 K), which are better than the values of most ternary chalcogenides (e.g., CuFeS_2_, Cu_3_SbSe_3_), including the recent, two-phase Cu_3_SbSe_4_–SnSe system [40], comparable to pure-phase copper chalcogenides and slightly worse than the recent Cu_12_Sb_4_S_13_ tetrahedrite obtained by Baláž et al. [41]. Therefore, it can be stated that the present approach makes it possible to obtain highly functional, low-temperature thermoelectric materials, characterized by excellent stability; greatly outperforming the typical phase-pure materials in this regard.

## 4. Conclusions

In the present study, an alternative way of improving the stability of copper chalcogenide-based thermoelectric materials is documented. Based on the combined theoretical and experimental studies, it is shown that the reduction of excessive Cu ion migration can be achieved by obtaining a mixture of stable binary and ternary Cu-Sb-Ch phases. Room-temperature structural and micro-structural investigations (XRD, SEM) confirm the presence of the composite materials, composed of orthorhombic Cu_1.97_S and secondary cubic Cu_12_Sb_4_S_13_ tetrahedrite structures and the mixture of two binary phases (Cu_1.8_Se, Cu_2_Se) with secondary precipitates of the diamond-like orthorhombic Cu_3_SbSe_3_ phase for sulfur- and selenium-rich samples, respectively. Secondary phases appear to precipitate mainly near the grain boundaries in the form of fibers or plates, significantly improving the stability of these materials, which was demonstrated on the basis of the cyclic measurements of transport properties. The investigated materials are characterized by electrical conductivity at the level of 10^4^ S·m^−1^ (S-rich), and 10^5^ (Se-rich) S·m^−1^, and Seebeck coefficient at the level of 60–100 μV·K^−1^. However, most importantly, the materials are characterized by the almost constant dependence of these transport properties as a function of temperature between subsequent thermal cycles, indicating good stability under working conditions. Unfortunately, the increased amount of the secondary phases also enhances the thermal conductivity values in comparison to the individual components of the materials. Thermoelectric figure-of-merit (ZT), calculated on the basis of electrical and thermal conductivity, as well as Seebeck coefficient for the presented materials with the smallest concentration of the ternary phase, reaches comparable levels to the pure-phase Cu_2−x_Ch compounds (in the considered temperature range). For higher concentrations of the secondary phases, the ZT values increase in the case of the sulfur-rich series and decrease in the case of the selenium-rich series. The presented approach makes it possible to obtain highly functional thermoelectric materials, with stability greatly beyond the capabilities of conventional, phase-pure systems.

## Figures and Tables

**Table 1:** Nominal compositions and densities of the synthesized samples and their abbreviations.

	Abbreviation	Nominal Cu_2_Ch Content [weight %]	Nominal Cu_12_Sb_4_Ch_13_ Content [weight %]	Density of the Samples [gcm^−1^]
Sulfides	CSS1	99% Cu_2_S	1% Cu_12_Sb_4_Se_13_	5.48
	CSS5	95% Cu_2_S	5% Cu_12_Sb_4_Se_13_	5.43
	CSS10	90% Cu_2_S	10% Cu_12_Sb_4_Se_13_	5.39
Selenides	CSE1	99% Cu_2_Se	1% Cu_3_SbSe_3_	6.70
	CSE5	95% Cu_2_Se	5% Cu_3_SbSe_3_	6.64
	CSE10	90% Cu_2_Se	10% Cu_3_SbSe_3_	6.57

**Table 2:** Defect formation energies and enthalpy of creation determined on the basis of optimized model structures and Equations (2)–(4).

Chemical Composition	Defect Formation Energy [eV]	Enthalpy of Creation [eV]
Cu_2_S cubic	−1.538 (single Cu vacancy)	−1.434
Cu_2_Se cubic	−3.894 (single Cu vacancy)	−0.213
Cu_12_Sb_4_S_13_	-	−6.826
Cu_12_Sb_4_Se_13_	-	−3.864
Cu_1.97_Sb_0.03_S	1.480	-
Cu_1.94_Sb_0.03_S	0.763	-
Cu_1.94_Sb_0.06_S	1.599	-
Cu_1.85_Sb_0.05_S	0.319	-
Cu_1.88_Sb_0.12_S_v1 (Figure 2a)	3.056	-
Cu_1.88_Sb_0.12_S_v2 (Figure 2b)	1.133	-
Cu_1.81_Sb_0.19_S_v1	1.363	-
Cu_1.81_Sb_0.19_S_v2	0.812	-
Cu_1.75_Sb_0.25_S_v1 (Figure 2c)	2.867	-
Cu_1.75_Sb_0.25_S_v2 (Figure 2d)	0.938	-
Cu_1.75_Sb_0.25_S_v3 (Figure 2e)	0.324	-
Cu_1.97_Sb_0.03_Se	−2.479	-
Cu_1.94_Sb_0.03_Se	−1.079	-
Cu_1.94_Sb_0.06_Se	1.638	-
Cu_1.85_Sb_0.05_Se	0.400	-
Cu_1.88_Sb_0.12_Se_v1 (Figure 2a)	3.997	-
Cu_1.88_Sb_0.12_Se_v2 (Figure 2b)	−0.324	-
Cu_1.81_Sb_0.19_Se_v1	0.811	-
Cu_1.81_Sb_0.19_Se_v2	0.575	-
Cu_1.75_Sb_0.25_Se_v1 (Figure 2c)	2.810	-
Cu_1.75_Sb_0.25_Se_v2 (Figure 2d)	2.586	-
Cu_1.75_Sb_0.25_Se_v3 (Figure 2e)	−0.287	-

## Data Availability

Data is contained within the article or Supplementary Materials.

## Supplementary Materials

The following are available online at https://www.mdpi.com/article/10.3390/ma14102635/s1, Figure S1. Optimized model structures: (a) Cu1.97Sb0.03Ch, (b) Cu1.94Sb0.03Ch, (c) Cu1.94Sb0.06Ch, (d) Cu1.85Sb0.05Ch, (e) Cu1.81Sb0.19Ch_v1, (f) Cu1.81Sb0.19Ch_v2.
