# Supplementary material for: Copper Chalcogenide–Copper Tetrahedrite Composites—A New Concept for Stable Thermoelectric Materials Based on the Chalcogenide System
Source: Materials (Basel). 2021 May 18;14(10):2635. doi: 10.3390/ma14102635 (PMC8157852; doi:10.3390/ma14102635)
Supplement: Supplementary file 1 [file materials-14-02635-s001.zip › materials-1201772-supplementary.pdf]

materials

Supplementary Materials

Copper Chalcogenide–Copper Tetrahedrite Composites—A New Concept for Stable Thermoelectric Materials Based on the Chalcogenide System

Andrzej Mikuła *, Krzysztof Mars, Paweł Nieroda and Paweł Rutkowski

Faculty of Materials Science and Ceramics, AGH University of Science and Technology, Al. Mickiewicza 30, 30-059 Krakow, Poland; kmars@agh.edu.pl (K.M.); pnieroda@agh.edu.pl (P.N.); pawelr@agh.edu.pl (P.R.)
* Correspondence: amikula@agh.edu.pl

Citation: Mikuła, A.; Mars, K.; Nieroda, P.; Rutkowski, P. Copper Chalcogenide-Copper Tetrahedrite Composites—A New Concept for Stable Thermoelectric Materials Based on Chalcogenide System. *Materials* **2021**, *14*, 2635. https://doi.org/10.3390/ma14102635

Academic Editor: Victor M. Prida

Received: 13 April 2021
Accepted: 14 May 2021
Published: 18 May 2021

Publisher's Note: MDPI stays neutral with regard to jurisdictional claims in published maps and institutional affiliations.

Copyright: © 2021 by the authors. Submitted for possible open access publication under the terms and conditions of the Creative Commons Attribution (CC BY) license (http://creativecommons.org/licenses/by/4.0/).

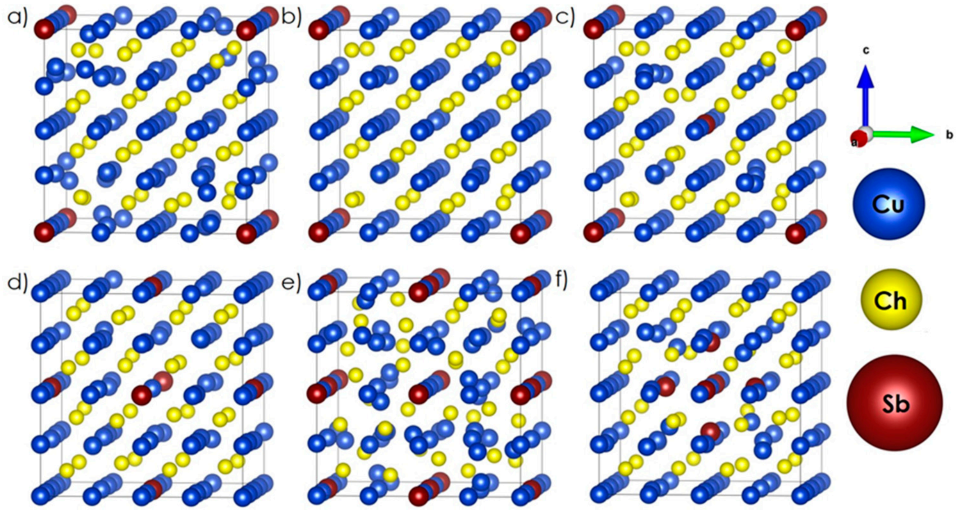

Figure S1. Optimized model structures: (**a**) Cu1.97Sb0.03Ch, (**b**) Cu1.94Sb0.03Ch, (**c**) Cu1.94Sb0.06Ch, (**d**) Cu1.85Sb0.05Ch, (**e**) Cu1.81Sb0.19Ch_v1, (**f**) Cu1.81Sb0.19Ch_v2.
